# Crystal structure of guanosine 5′-monophosphate synthetase from the thermophilic bacterium *Thermus thermophilus* HB8

**DOI:** 10.1107/S2053230X2400877X

**Published:** 2024-09-18

**Authors:** Naoki Nemoto, Seiki Baba, Gota Kawai, Gen-ichi Sampei

**Affiliations:** a Faculty of Advanced Engineering Chiba Institute of Technology Narashino Chiba275-0016 Japan; b RIKEN SPring-8 Center, Harima Institute, 1-1-1 Kouto, Sayo, Hyogo679-5148, Japan; c Graduate School of Informatics and Engineering The University of Electro-Communications 1-5-1 Chofugaoka Chofu Tokyo182-8585 Japan; Osaka University, Japan

**Keywords:** *AlphaFold*2, guanosine monophosphate synthetase, molecular-dynamics simulations, purine nucleotide biosynthetic pathway, thermophiles

## Abstract

Guanosine 5′-monophosphate (GMP) synthetase (GuaA) catalyzes the last step of GMP synthesis in the purine nucleotide biosynthetic pathway. This enzyme catalyzes a reaction in which xanthine 5′-monophosphate (XMP) is converted to GMP in the presence of Gln and ATP through an adenyl-XMP intermediate. A structure of an XMP-bound form of GuaA from the domain Bacteria has not yet been determined. In this study, the crystal structure of an XMP-bound form of GuaA from the thermophilic bacterium *Thermus thermophilus* HB8 (*Tt*GuaA) was determined at a resolution of 2.20 Å and that of an apo form of *Tt*GuaA was determined at 2.10 Å resolution. *Tt*GuaA forms a homodimer, and the monomer is composed of three domains, which is a typical structure for GuaA. Disordered regions in the crystal structure were obtained from the *AlphaFold*2-predicted model structure, and a model with substrates (Gln, XMP and ATP) was constructed for molecular-dynamics (MD) simulations. The structural fluctuations of the *Tt*GuaA dimer as well as the interactions between the active-site residues were analyzed by MD simulations.

## Abstract 2

Guanosine 5′-monophosphate (GMP) synthetase (GuaA) catalyzes the last step of GMP synthesis in the purine nucleotide biosynthetic pathway. In this study, the crystal structure of an XMP-bound form of GuaA from the thermophilic bacterium *T. thermophilus* HB8 was determined at a resolution of 2.20 Å and that of an apo form was determined at 2.10 Å resolution.

## 1. Introduction

**Figure 1:**
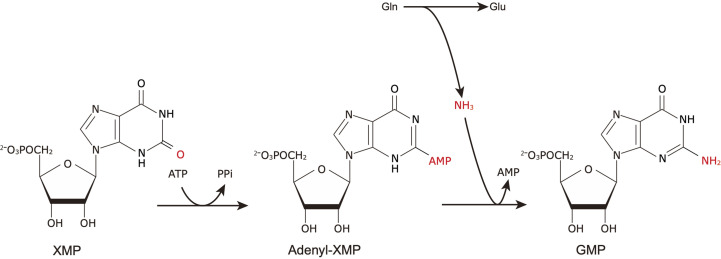
Reaction scheme of GuaA in the purine synthetic pathway.

Guanosine 5′-monophosphate (GMP) synthetase (GuaA) catalyzes the final step of GMP synthesis in the purine nucleotide biosynthetic pathway (Hartman & Buchanan, 1959 ▸; Miller & Buchanan, 1962 ▸). This enzyme catalyzes a reaction in which xanthine 5′-monophosphate (XMP) is converted to GMP in the presence of Gln and ATP through an adenyl-XMP intermediate (Fig. 1 ▸; Fukuyama, 1966 ▸).

Crystal structures of GuaA have so far been determined from several organisms. GuaA forms a homodimer. The monomer consists of three domains: a class I glutamine amidotransferase (GATase) domain, an ATP pyrophos­phatase (ATPPase) domain and a dimerization domain. GATase hydrolyzes glutamine to generate glutamate and ammonia. The GATase domain has a catalytic triad formed by conserved Cys–His–Glu residues. ATPPase adenylates XMP to form an adenyl-XMP intermediate in the presence of Mg^2+^, XMP and ATP. Adenyl-XMP is aminated by ammonia to form GMP. During the reaction, the ammonia molecule needs to move from the GATase domain to the ATPPase domain.

The first crystal structure of GuaA was determined for the *Escherichia coli* enzyme in complex with AMP and PP_i_ (PDB entry 1gpm; Tesmer *et al.*, 1996 ▸). The crystal structure of GuaA from *Plasmodium falciparum* in complex with glutamine has been determined (PDB entry 4wio; Ballut *et al.*, 2015 ▸). Crystal structures of GuaA–XMP complexes have been determined for the enzymes from *Homo sapiens* (PDB entry 2vxo; Welin *et al.*, 2013 ▸), *P. falciparum* (PDB entry 3uow; Structural Genomics Consortium, unpublished work) and *Methanococcus jannaschii* (PDB entry 6jp9; Shivakumaraswamy *et al.*, 2022 ▸); however, there are no reports of GuaA–XMP complexes of enzymes from the domain Bacteria.

Here, we report crystal structures of GuaA from *Thermus thermophilus* HB8 in the apo form and in complex with XMP (*Tt*GuaA–XMP). *T. thermophilus* HB8 is a thermophilic bacterium and the structures of many proteins related to the purine biosynthetic pathway derived from *T. thermophilus* have previously been determined (Sampei *et al.*, 2023 ▸; Nemoto *et al.*, 2023 ▸). This is the first crystal structure of an XMP-complexed GuaA from the domain Bacteria to be determined. Disordered regions in the crystal structure were obtained from an *AlphaFold*2-predicted model structure, and a model with substrates (Gln, XMP and ATP) was constructed for molecular-dynamics (MD) simulations. The structural fluctuations of the *Tt*GuaA dimer as well as the interactions between the active-site residues were analyzed by MD simulations.

## 2. Materials and methods

### 2.1. Cloning, expression, purification and crystallization of *Tt*GuaA

Protein sample preparation, crystallization and diffraction measurements were performed within the Structural-Biological Whole Cell Project of *Thermus thermophilus* HB8 (https://www.thermus.org/). The TTHA1552 gene was amplified by PCR using *T. thermophilus* HB8 genomic DNA as the template and ligated into the expression vector pET-11a. *Escherichia coli* strain Rosetta(DE3) cells carrying the plasmid were grown and the *Tt*GuaA protein was obtained from the cell extract. After heat treatment at 70°C for 10 min, the *Tt*GuaA protein was purified by hydrophobic interaction (Resource ISO 6 ml column; Cytiva), anion-exchange (Resource Q 6 ml column; Cytiva), hydroxyapatite (CHT2 2 ml column; Bio-Rad) and gel-filtration (HiLoad 16/60 Superdex 75 pg column; Cytiva) column chromatography. Finally, the protein sample was obtained as a 9.08 mg ml^−1^ solution in 20 m*M* Tris–HCl pH 8.0, 150 m*M* NaCl, 1 m*M* DTT. 10.9 mg of purified protein was obtained from 32 g of *E. coli* cells. Macromolecule-production information is summarized in Table 1 ▸.

The crystal of *Tt*GuaA in the apo form was obtained by the hanging-drop vapor-diffusion method at 20°C using reservoir solution consisting of 0.1 *M* sodium acetate tri­hydrate pH 4.6, 1.4 *M* NaCl. The crystal of the *Tt*GuaA–XMP complex was obtained by the sitting-drop vapor-diffusion method at 20°C using reservoir solution consisting of 0.1 *M* sodium acetate trihydrate pH 4.4, 1.2 *M* NaCl, 10 m*M* XMP (Table 2 ▸).

### 2.2. Data collection and structure determination

X-ray intensity data were collected on beamlines BL41XU and BL26B1 at SPring-8. The collected diffraction data were processed using *HKL*-2000 (Otwinowski & Minor, 1997 ▸). Initially, a multiple-wavelength anomalous diffraction (MAD) data set was collected using selenomethionine-labeled *Tt*GuaA for phase determination. The structure of apo *Tt*GuaA was determined using native *Tt*GuaA with phase information obtained from the MAD data. In the case of *Tt*GuaA–XMP, the structure was determined by collecting edge data from selenomethionyl protein crystals, followed by molecular replacement using the phase information from the apo *Tt*GuaA structure with *MOLREP* (Vagin & Teplyakov, 2010 ▸) in the *CCP*4 suite (Agirre *et al.*, 2023 ▸). Both structures were refined with *CNS* (version 1.1; Brünger *et al.*, 1998 ▸). The structures were deposited in the Protein Data Bank with PDB codes 2ywb (apo *Tt*GuaA) and 2ywc (*Tt*GuaA–XMP). Data-collection and refinement statistics are shown in Tables 3 ▸ and 4 ▸, respectively.

### 2.3. Preparation of the full-length model with ligands

To perform MD simulations, a full-length model of the *Tt*GuaA dimer (subunits *A* and *B*) with ligands was prepared. Amino-acid residues of disordered regions in both subunits of *Tt*GuaA–XMP (residues 324–339 and 433–444 of subunit *A* and residues 323–346 and 433–440 of subunit *B*) were superposed and supplemented with a structure predicted by *AlphaFold*2 (GuaA from *T. thermophilus* HB8; model ID AF-A0A3P4APK1-F1-model_v4; Jumper *et al.*, 2021 ▸; Varadi *et al.*, 2022 ▸). The r.m.s.d. between the *AlphaFold*2 model and the crystal structure of apo *Tt*GuaA was 0.498 Å and the r.m.s.d. between the *AlphaFold*2 model and *Tt*GuaA–XMP was 0.531 Å. The r.m.s.d.s for the pairs of amino-acid residues of the stem loops leading to the added loops between the *AlphaFold*2 model and *Tt*GuaA–XMP were 0.607 Å (322–323 and 341–342 in the D1 region) and 0.760 Å (431–432 and 455–456 in the D2 region), respectively. The structure of ligand-bound *Tt*GuaA was constructed using information from co-crystal structures with the ligands. *Tt*GuaA and ligand-bound GuaA were superposed by *Chimera* (Pettersen *et al.*, 2004 ▸) and the values of the coordinates of the ligand were inserted into the coordinates of *Tt*GuaA–XMP. Each ligand was added to both molecules of the *Tt*GuaA–XMP dimer. AMP, POP (PP_i_) and Mg^2+^ in PDB entry 1gpm (Tesmer *et al.*, 1996 ▸) were used as templates for ATP and Mg^2+^. Similarly, the glutamine in PDB entry 4wio (Ballut *et al.*, 2015 ▸) was used as a template.

### 2.4. MD simulations

MD simulations were performed with *AMBER*22 (Case *et al.*, 2022 ▸) as described previously (Nemoto *et al.*, 2023 ▸). A productive simulation of 300 ns (300 000 000 steps) in a constant volume without positional restraints was performed three times with randomized initial velocities. To neutralize the system, 28 sodium ions were added, followed by 44 903 water molecules.

## 3. Results

### 3.1. Overall structure of *Tt*GuaA

**Figure 2:**
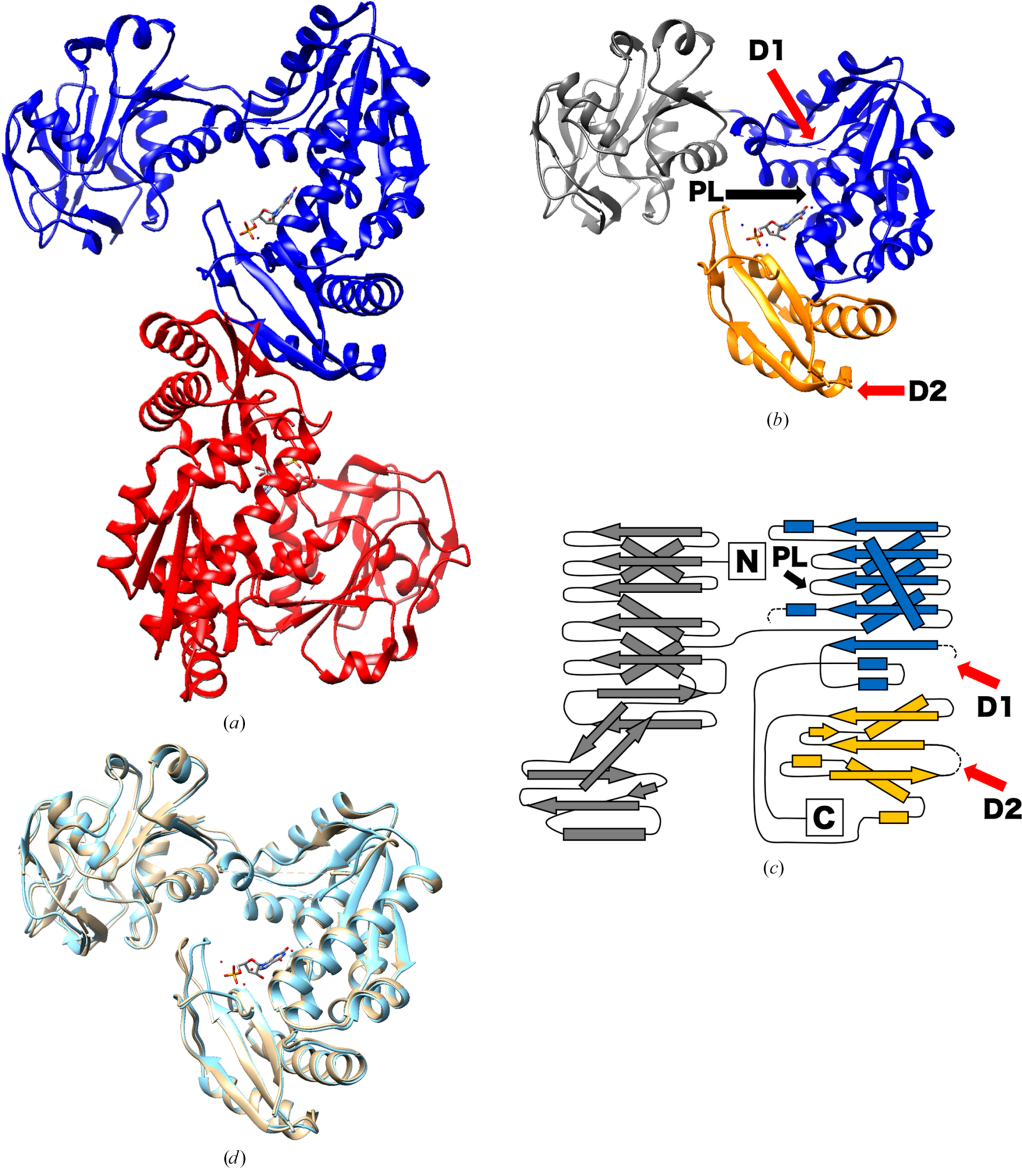
Crystal structure of *Tt*GuaA. (*a*) Ribbon diagram of the *Tt*GuaA homodimer in complex with XMP. Subunit *A* is shown in blue and subunit *B* in red. (*b*) The *Tt*GuaA monomer in complex with XMP (*Tt*GuaA–XMP). The N-terminal glutamine amidotransferase (GATase) domain (residues 1–188) is shown in gray, the ATP pyrophosphatase (ATPPase) domain (residues 189–390) in blue and the C-terminal dimerization domain (residues 391–503) in orange. The two disordered regions 324–339 and 433–444, labeled D1 and D2, respectively, are indicated by red arrows. The P-loop (217–222), labeled PL, is indicated by a black arrow. (*c*) Schematic drawing of the secondary structure of subunit *A* of *Tt*GuaA–XMP. The meanings of the colors are the same as in (*b*). The disordered regions D1 and D2 are indicated by dashed lines and red arrows, respectively. The P-loop, labeled PL, is indicated by a black arrow. (*d*) Superposition of the *Tt*GuaA proteins. The apo form of *Tt*GuaA is shown in tan and *Tt*GuaA–XMP in shown in cyan.

The crystal structures of the apo and XMP-complexed forms of *Tt*GuaA were determined at resolutions of 2.10 and 2.20 Å, respectively. The asymmetric unit of both *Tt*GuaA crystals contained four *Tt*GuaA molecules (chains *A*–*D*). *Tt*GuaA was estimated to form a homodimer by size-exclusion chromatography. Chains *A* and *B* and chains *C* and *D* formed homodimers (Fig. 2 ▸*a*). *Tt*GuaA is composed of three domains: a GATase domain (residues 1–188), an ATPPase domain (residues 189–390) and a dimerization domain (residues 391–503) (Figs. 2 ▸*b* and 2 ▸*c*). No electron density was observed for residues 324–339 and 433–444 of subunit *A* and for residues 323–346 and 433–444 of subunit *B* in both crystal structures (Figs. 2 ▸*b* and 2 ▸*c*). In addition, residues 95–97 of subunit *A* and residues 96–99 of subunit *B* were disordered in apo *Tt*GuaA. In *Tt*GuaA–XMP, one XMP molecule was bound to each monomer. The XMP molecule was located 40 Å away from the active site of the GATase domain, showing a relationship similar to that observed in other structures of GuaA. No tunnels that can efficiently transfer ammonia to XMP were observed in the crystal structure.

The r.m.s.d. between the crystal structures of apo *Tt*GuaA and *Tt*GuaA–XMP was 0.578 Å. Differences were only observed at the position of the loop near the substrate XMP (Fig. 2 ▸*d*).

A comparison of *Tt*GuaA with GuaA from *E. coli* (*Ec*GuaA) showed that the sequence identity between *Tt*GuaA and *Ec*GuaA was 51.3% and the r.m.s.d. between the crystal structure of *Tt*GuaA subunit *A* and that of *Ec*GuaA subunit *A* was 1.135 Å (for apo *Tt*GuaA) or 1.161 Å (for *Tt*GuaA–XMP) (Supplementary Figs. S1*a* and S1*b*). The dimerization domain of *Tt*GuaA has a more compact structure than that of *Ec*GuaA. In *Tt*GuaA, Arg465 and Asp472, as well as Arg487 and Asp491, formed salt bridges between the dimerization domains. In *Ec*GuaA, residues corresponding to Arg487 and Asp491 formed a salt bridge between Arg509 and Asp513, whereas the residues corresponding to Arg465 and Asp472 of *Tt*GuaA were different amino acids (His487 and Gly494) and did not form a salt bridge. The amino acids corresponding to Arg465 and Asp472 of GuaA in the thermophilic bacteria *Thermotoga maritima* and *Aquifex aeolicus* formed pairs consisting of Arg and Asp and of Lys and Asp, respectively, which potentially form salt bridges. The compactness of the dimerization domain and the formation of salt bridges were considered to potentially contribute to the thermostability of these thermophilic enzymes.

### 3.2. XMP-binding site of *Tt*GuaA

The XMP molecule is bound in the active site located between the ATPPase domain and the dimerization domain (Fig. 2 ▸). The xanthine base is surrounded by a conserved proline-rich region (Pro382, Gly383 and Pro384), and a side-chain atom of Arg288 interacts with O6 (subunits *A* and *B*) and N7 (subunit *B*) of the xanthine base (Supplementary Fig. S2). The ribose moiety interacts with a side-chain atom of Gln424. The phosphate moiety of XMP interacts with a side-chain atom of Lys495 and main-chain atoms of Ile500 and Glu501.

Crystal structures of GuaA in complex with XMP have been determined for the enzymes from *H. sapiens* (*Hs*GuaA; PDB entry 2vxo; Welin *et al.*, 2013 ▸) and *P. falciparum* (*Pf*GuaA; PDB entry 3uow; Structural Genomics Consortium, unpublished work). A crystal structure of the ATPPase domain in complex with XMP has been determined for GuaA from *M. jannaschii* (*Mj*GuaA; PDB entry 6jp9; Shivakumaraswamy *et al.*, 2022 ▸). Comparison of the crystal structure of the XMP-binding site of *Tt*GuaA with those of these proteins revealed that the XMP-recognition residues were highly conserved, with the exception of Thr690 in *Hs*GuaA, which corresponds to Ile500 in *Tt*GuaA.

### 3.3. MD simulations

**Figure 3:**
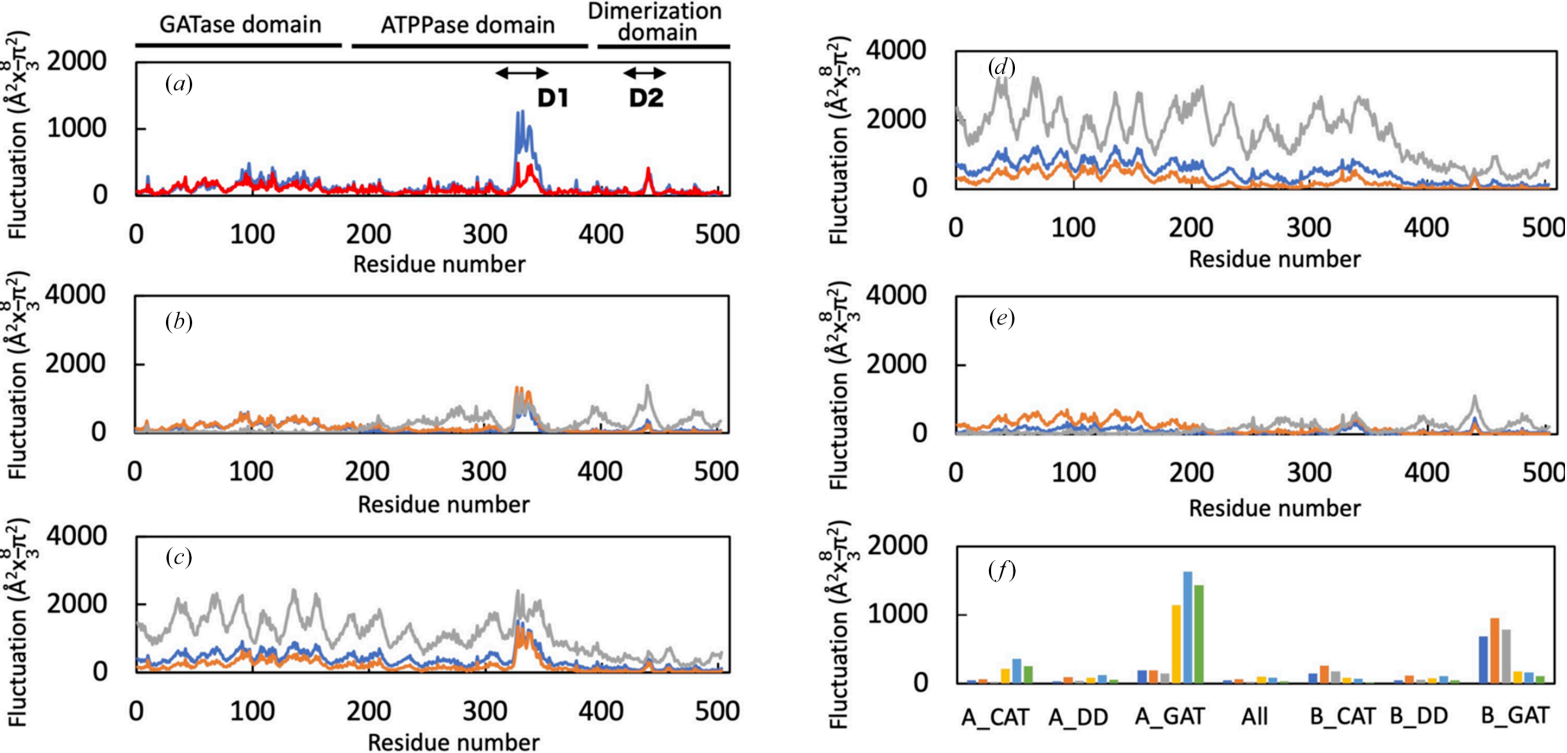
Conformational fluctuations of the *Tt*GuaA model deduced from analysis of the MD simulations. Atomic fluctuations are represented for each amino-acid residue as the scale of the *B* factor. (*a*) Fluctuations of the whole structure of subunits *A* (blue) and *B* (red). Two disordered regions (D1 and D2) are indicated by bidirectional arrows. (*b*) The fluctuations of subunit *A* when the GATase domain (1–185) of subunit *A* (gray), the ATPPase domain (192–389) of subunit *A* (blue) and the dimerization domain (396–503) of subunit *A* (orange) were fixed. (*c*) The fluctuations of subunit *A* when the GATase domain of subunit *B* (gray), the ATPPase domain of subunit *B* (blue) and the dimerization domain of subunit *B* (orange) were fixed. (*d*) The fluctuations of subunit *B* when the GATase domain of subunit *A* (gray), the ATPPase domain of subunit *A* (blue) and the dimerization domain of subunit *A* (orange) were fixed. (*e*) The fluctuations of subunit *B* when the GATase domain of subunit *B* (gray), the ATPPase domain of subunit *B* (blue) and the dimerization domain of subunit *B* (orange) were fixed. (*f*) The fluctuations of the ligands and Mg^2+^ ion when subunits *A* and *B* (All) or three domains (GATase domain, GAT; ATPPase domain, CAT; dimerization domain, DD) of each subunit were fixed. The bars of the fluctuations are colored as follows: XMP in subunit *A*, blue; ATP in subunit *A*, orange; Mg^2+^ in subunit *A*, gray; XMP in subunit *B*, yellow; ATP in subunit *B*, cyan; Mg^2+^ in subunit *B*, green.

The structural fluctuations of amino-acid residues in the full-length model of *Tt*GuaA with the substrates Gln, XMP and ATP were investigated by MD simulations. As shown in Fig. 3 ▸, the fluctuations of disordered regions (D1 and D2) in the crystal structure of *Tt*GuaA were larger than those of the other regions. The region with particularly large fluctuations is a lid loop (D1, 323–340), which is close to the active site. The lid loop was disordered in most GuaA structures from other organisms. By partially utilizing the *AlphaFold*2 model for the disordered regions of the crystal structure, the large movements of the loops and the reasons for the disorder were confirmed. However, a different method of verification is needed to discuss the influence of the substrate.

The fluctuations of the GATase domain were larger than those of the ATPPase and dimerization domains, except for the D1 and D2 regions (Fig. 3 ▸*a*). When each domain of subunits *A* and *B* was fixed and its fluctuations were analyzed, it was confirmed that the GATase domain moves independently of the other domains in both subunits. It was also shown that the ATPPase and dimerization domains move in conjunction with each other in subunits *A* and *B* (Figs. 3 ▸*b*–3 ▸*f*).

Substrate-binding residues were confirmed using the structure at 45 ns during the MD simulations (Supplementary Fig. S3). Cys78, His164 and Glu166 form a catalytic triad. In the structure of the *Tt*GuaA model at 45 ns, the glutamine interacts with six residues: Ser9, Gly51, Tyr79, Tyr100, Ser125 and His164 (Supplementary Fig. S3*a*). The other two residues of the catalytic triad did not directly interact with the glutamine, although these residues were located close to the glutamine. The fluctuations of the glutamine were larger than those of the entire structure, and the glutamine showed no interactions with amino-acid residues after 70.8 ns.

The phosphate groups of ATP were surrounded by the P-loop (217–222) and were recognized by Ser217, Asp221, Ser222, Lys359 and Arg378 (Supplementary Fig. S3*b*). The ribose of ATP was recognized by Gly315 and the adenine base of ATP was recognized by His336.

## 4. Discussion

The MD simulation results showed that the GATase domain had larger fluctuations than the other domains. In some species from the domain Archaea, such as *Pyrococcus horikoshii* and *M. jannaschii*, the GATase domain is encoded by a different gene to that for the ATPPase and dimerization domains and is composed of an independent polypeptide (Maruoka *et al.*, 2010 ▸; Shivakumaraswamy *et al.*, 2022 ▸). Although movements were observed in the GATase domain, the substrate Gln, which was located at the active site of the GATase domain, did not approach XMP, which was located at the active site of the ATPPase domain and remained 40 Å away. No rotation of the GATase domain, as reported for GuaA from *P. falciparum* (Ballut *et al.*, 2015 ▸), was observed. Furthermore, the presence of a tunnel through which ammonia moves directly inside the protein molecule could not be confirmed. There are three reaction steps in the purine nucleotide synthetic pathway that utilize ammonia generated from Gln as catalyzed by GATase. The enzymes involved in these reaction steps are glutamine phosphoribosylpyrophos­phate (PRPP) amidotransferase (PurF), formylglycinamide ribonucleotide (FGAR) amidotransferase (the PurLSQ complex) and GuaA. PurF catalyzes the reaction step in which the pyrophosphate in PRPP binds to ammonia derived from Gln (Chen *et al.*, 1997 ▸; Wang *et al.*, 2019 ▸). The PurLSQ complex catalyzes the reaction step to produce formylglycin­amidine ribonucleotide (FGAM). PurQ is equivalent to the GATase domain (Morar *et al.*, 2006 ▸; Suzuki *et al.*, 2012 ▸). The GATase domains of GuaA and PurQ have similarities in their three-dimensional structures; however, they show no similarity to the GATase domain of PurF.

**Figure 4:**
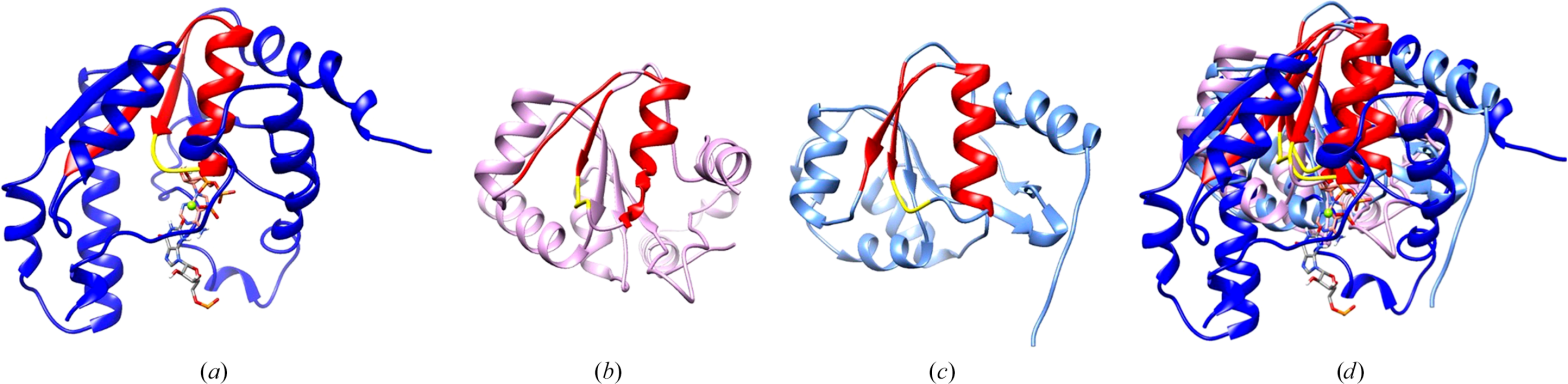
Structural comparison of adenylation domains. The common motif is shown in red. The P-loop is shown in yellow. (*a*) The ATPPase domain (residues 189–390) of *Tt*GuaA with the XMP and ATP model structure. The C atoms of XMP and ATP are shown in silver and orange, respectively. (*b*) The adenylation domain of firefly luciferase (residues 200–355 of PDB entry 1lci). (*c*) The adenylation domain of acyl-CoA synthetase from *T. thermophilus* (residues 200–360 of PDB entry 1ult). (*d*) Superposition of the three adenylation domains.

The ATPPase domain activates the substrate XMP by adenylation for reactions to proceed. GuaA belongs to the PP-loop ATP pyrophosphatase family (Fellner *et al.*, 2018 ▸). The characteristic feature of this family is that its members possess a P-loop motif that is used for ATP binding and substrate adenylation. This family includes arginine synthetase, argininosuccinate synthetase, tRNA 4-thiouridylase and GuaA. The ATPPase domain of *Tt*GuaA was structurally compared with the adenylation domains of acyl-CoA synthetase and firefly luciferase. As a result, acyl-CoA synthetase and firefly luciferase were found to activate substrates by adenylation for reactions to proceed and they are considered to be evolutionarily related (Conti *et al.*, 1996 ▸; Oba *et al.*, 2020 ▸). Although the sequence similarities of the adenylation domains of *Tt*GuaA and these enzymes were shown to be low, they have a common motif (Fig. 4 ▸). The common motif is the Rossmann-like fold (β–P-loop–α–β), although one of the β-sheets forms a loop in firefly luciferase. These enzymes that activate substrates by adenylation are considered to have a common ancestral motif.

In the purine nucleotide synthetic pathway, glycinamide ribonucleotide (GAR) synthetase (PurD) adds amino groups to phosphorylated substrates. PurD catalyzes the activation of glycine by phosphorylation by ATP and the formation of GAR by the binding of phosphorylated glycine to the amino group of phosphoribosylamine (Sampei *et al.*, 2010 ▸; Yamamoto *et al.*, 2022 ▸). Even within the same purine nucleotide synthesis pathway, the activation of substrates by ATP for amination can occur via phosphorylation or adenylation. The whole-cell project on *T. thermophilus* (Yokoyama *et al.*, 2000 ▸; Iino *et al.*, 2008 ▸; Bessho, 2023 ▸) has led to the accumulation of information on proteins from *T. thermophilus* and the present study has contributed to this. The information on the structure of *Tt*GuaA will be accessible in the new *Thermus* database called ThermusQ (https://www.thermusq.net/; Hijikata *et al.*, 2023 ▸).

## 5. Related literature

The following reference is cited in the supporting information for this article: Laskowski & Swindells (2011 ▸).

## Supplementary Material

PDB reference: GMP synthetase from *Thermus thermophilus*, 2ywb

PDB reference: complex with XMP, 2ywc

revised Supplementary Figures. DOI: 10.1107/S2053230X2400877X/nw5127sup1.pdf

## Figures and Tables

**Table 1:** Macromolecule-production information of *Tt*GuaA

Source organism	*Thermus thermophilus* (strain HB8)
DNA source	Genomic DNA of *T. thermophilus* HB8
Expression vector	pET-11a
Expression host	*E. coli* strain Rosetta(DE3)
Complete amino-acid sequence of the construct produced	MVLVLDFGSQYTRLIARRLRELRAFSLILPGDAPLEEVLKHRPQALILSGGPRSVFDPDAPRPDPRLFSSGLPLLGICYGMQLLAQELGGRVERAGRAEYGKALLTRHEGPLFRGLEGEVQVWMSHQDAVTAPPPGWRVVAETEENPVAAIASPDGRAYGVQFHPEVAHTPKGMQILENFLELAGVKRDWTPEHVLEELLREVRERAGKDRVLLAVSGGVDSSTLALLLAKAGVDHLAVFVDHGLLRLGEREEVEGALRALGVNLLVVDAKERFLKALKGVEDPEEKRKIIGREFVAAFSQVARERGPFRFLAQGTLYPDVIESAGGHGAAKIKSHHNVGGLPEDLEFELLEPFRLLFKDEVRELALLLGLPDTLRLRHPFPGPGLAVRVLGEVTEERLEILRRADDIFTSLLREWGLYEKVAQALAVLTPVRSVGVAGDERKYGYVLALRAVTTEDFMTADWARLPLEFLDEAARRITRRVPEIGRVVYDLTSKPPATIEWE

**Table 2:** Crystallization conditions

	Apo *Tt*GuaA	*Tt*GuaA–XMP
Method	Vapor diffusion, hanging drop	Vapor diffusion, sitting drop
Plate type	Hampton Research 24-well plate	Hampton Research 24-well plate
Temperature (K)	293	293
Protein concentration (mg ml^−1^)	9.08	9.08
Buffer composition of protein solution	20 m*M* Tris–HCl pH 8.0, 150 m*M* NaCl, 1 m*M* DTT	20 m*M* Tris–HCl pH 8.0, 150 m*M* NaCl, 1 m*M* DTT
Composition of reservoir solution	0.1 *M* sodium acetate trihydrate pH 4.6, 1.4 *M* NaCl	0.1 *M* sodium acetate trihydrate pH 4.4, 1.2 *M* NaCl, 10 m*M* XMP
Volume and ratio of drop	1 µl, 1:1 ratio	1 µl, 1:1 ratio
Volume of reservoir (µl)	300	300

**Table 3:** Data collection and processing The values in parentheses are for the highest resolution shells: 2.18–2.10 Å for the apo-form crystal and 2.28–2.20 Å for the XMP-complex crystal.

	Apo *Tt*GuaA	*Tt*GuaA–XMP
Diffraction source	BL41XU, SPring-8	BL26B1, SPring-8
Wavelength (Å)	1.000	0.97891
Temperature (K)	100	100
Detector	MAR165	Jupiter210
Space group	*C*2	*C*2
*a*, *b*, *c* (Å)	140.949, 114.854, 160.033	142.580, 115.213, 159.384
α, β, γ (°)	90.0, 93.37, 90.0	90.0, 93.21, 90.0
Resolution range (Å)	50.00–2.10	50.00–2.20
No. of unique reflections	145950	126620
Completeness (%)	98.2 (94.7)	98.3 (97.4)
Multiplicity	3.8 (3.5)	5.5 (5.1)
〈*I*/σ(*I*)〉	16.4 (2.2)	23.1 (2.7)
*R*_r.i.m._ †	0.077 (0.486)	0.113 (0.891)
Overall *B* factor from Wilson plot (Å^2^)	25.0	25.4

† Estimated *R*_r.i.m._ = *R*_merge_[*N*/(*N* − 1)]^1/2^, where *N* is the data multiplicity.

**Table 4:** Structure refinement of *Tt*GuaA The values in parentheses are for the highest resolution shells: 2.23–2.10 Å for the apo-form crystal and 2.34–2.20 Å for the XMP-complex crystal.

	Apo *Tt*GuaA	*Tt*GuaA–XMP
Resolution range (Å)	43.28–2.10	31.82–2.20
Completeness (%)	91.3 (81.10)	87.9 (67.30)
σ Cutoff	*F* > 0.0σ(*F*)	*F* > 0.0σ(*F*)
No. of reflections, working set	135353 (18011)	125608 (26125)
No. of reflections, test set	13495 (1953)	21549 (2763)
Final *R*_cryst_	0.233 (0.314)	0.236 (0.407)
Final *R*_free_	0.272 (0.339)	0.278 (0.425)
No. of non-H atoms		
Protein	14709	14799
Ligand	—	96
Water	356	306
Total	15065	15201
R.m.s. deviations		
Bond lengths (Å)	0.006	0.007
Angles (°)	1.3	1.4
Average *B* factors (Å^2^)		
Main-chain atoms	45.49	46.98
Side-chain atoms	53.52	54.45
Ligand	—	55.21
Water atoms	41.48	41.28
Ramachandran plot		
Favored regions	1426 [91.3%]	1403 [89.3%]
Allowed regions	132 [8.5%]	165 [10.5%]
Outlier regions	4 [0.3%]	4 [0.3%]
