# Supplementary material for: Crystal structure of guanosine 5′-monophosphate synthetase from the thermophilic bacterium Thermus thermophilus HB8
Source: Acta Crystallogr F Struct Biol Commun. 2024 Sep 18;80(Pt 10):278–85. doi: 10.1107/S2053230X2400877X (PMC11448925; doi:10.1107/S2053230X2400877X)
Supplement: Supplementary file 1 [file f-80-00278-sup1.pdf]

STRUCTURAL BIOLOGY
COMMUNICATIONS

Volume 80 (2024)

Supporting information for article:

Crystal structure of guanosine 5′-monophosphate synthetase from the thermophilic bacterium *Thermus thermophilus* HB8

Naoki Nemoto, Seiki Baba, Gota Kawai and Gen-ichi Sampei

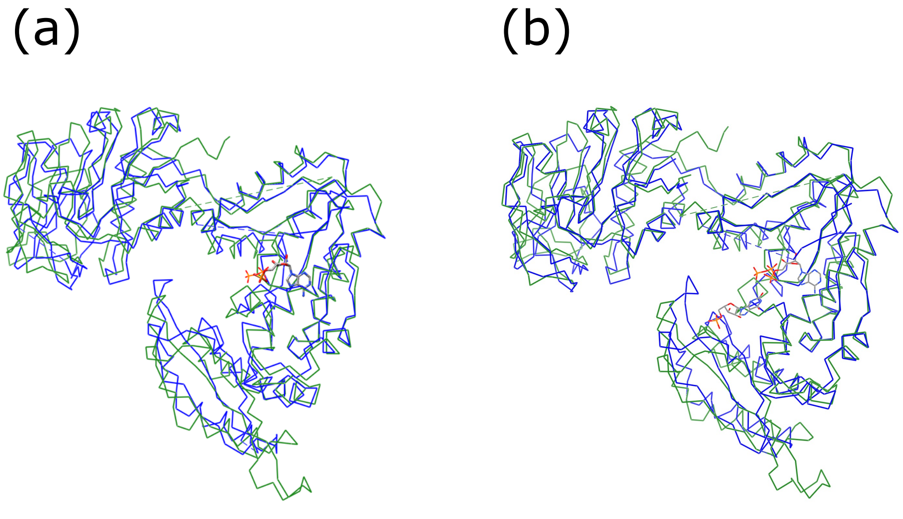

Supplementary Figure S1. Structure comparison of GuaA proteins.

(a) Superposition of *Tt*GuaA/apo and *Ec*GuaA. (b) Superposition of t *Tt*GuaA/XMP and *Ec*GuaA. *Tt*GuaA is in blue. The AMP-pyrophosphate-bound form of *Ec*GuaA (PDB ID 1gpm) is shown in green.

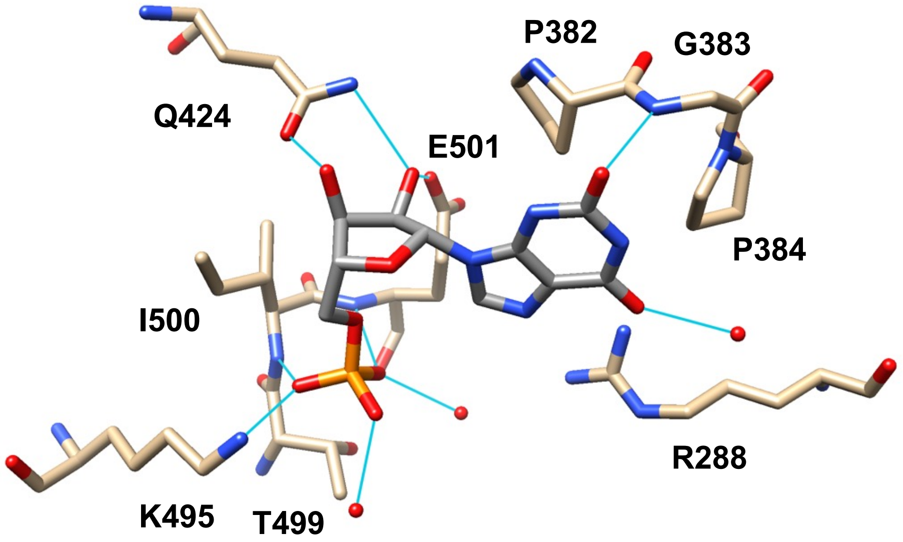

Supplementary Figure S2. Structure of XMP-binding site of *Tt*GuaA.

The active-site residues are shown for XMP. Water molecules are shown as red spheres. The pale blue lines indicate hydrogen bonds.

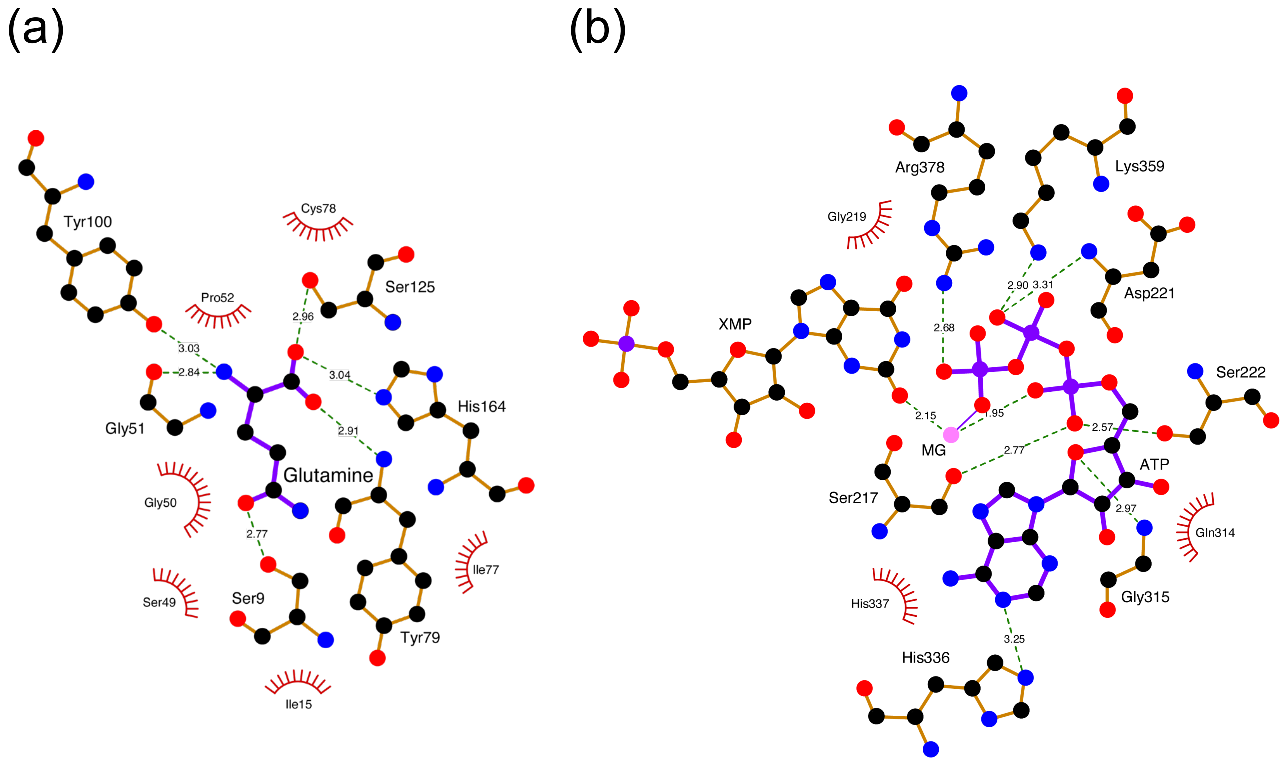

Supplementary Figure S3. Gln and ATP binding site of *Tt*GuaA model during MD simulation. Schematic diagram of the mechanism of recognition of (a) Gln and (b) ATP by *Tt*GuaA model during MD simulation at 45 ns. O atoms, N atoms, P atoms, and the $Mg2^{+}$ ion are shown in red, blue, purple, and pink, respectively. Residues that form hydrophobic contacts with the ligands are shown in red. Hydrogen bonds between the *Tt*GuaA model and the ligands are indicated by green dotted lines. The figure was prepared using LIGPLOT (Laskowski and Swindells, 2011)..

References

1. Laskowski, R. A. and Swindells, M. B. (2011). *J. Chem. Inf. Model.,* **51**, 2778-2786.
